# Clinical outcome of renal cancer patients who early interrupted immunotherapy due to serious immune-related adverse events. Meet-Uro 13 trial on behalf of the MeetUro investigators

**DOI:** 10.1186/s12967-021-03008-9

**Published:** 2021-08-03

**Authors:** Marco Stellato, Giuseppe Procopio, Ugo De Giorgi, Marco Maruzzo, Davide Bimbatti, Alessia Mennitto, Andrea Sbrana, Giandomenico Roviello, Chiara Casadei, Pierangela Sepe, Sandro Pignata, Daniele Santini

**Affiliations:** 1 Department of Medical Oncology, Campus Bio-Medico University of Rome, Via Alvaro del Portillo 200, 00128 Rome, Italy; 2 Medical Oncology Department, Fondazione IRCCS Istituto Nazionale dei Tumori, Milan, Italy; 3 IRCCS Istituto Romagnolo per lo Studio dei Tumori (IRST) Dino Amadori, Meldola, Italy; 4 Medical Oncology Unit 1, Department of Oncology, Istituto Oncologico Veneto IOV IRCCS, Padua, Italy; 5 Division of Oncology, University Hospital “Maggiore Della Carità”, Novara, Italy; 6 Department of Surgical, Medical and Molecular Pathology and Critical Area Medicine, University of Pisa, Pisa, Italy; 7 Department of Health Sciences, University of Florence, Florence, Italy; 8 Department of Urology and Gynecology, Istituto Nazionale Tumori IRCCS Fondazione “G. Pascale”, Naples, Italy

**Keywords:** Renal cell carcinoma, Immunotherapy, Immune-related adverse events

## Abstract

**Background:**

Severe immune-related Adverse Events (irAEs) develop in 10–27% of patients treated with Immune-Oncology (IO) [Powles (Lancet 391:748–757, 2018); Galsky (Lancet 395:1547–1557, 2020); Haanen (Ann Oncol 28:119–142, 2017)]. The aim of our study was to evaluate efficacy and clinical outcome of metastatic renal cell carcinoma (mRCC) patients who stopped Immune Checkpoint Inhibitors (ICIs) due to early Grade (G) 3-G4 irAEs.

**Methods:**

We retrospectively collected data from 204 mRCC patients treated with ICIs in 6 Italian referral centers adhering to the Meet-Uro group, between February 2017 and January 2020. To properly weight the results, patients who did not report early G3–G4 toxicities have been included as control group.

Primary endpoint was to evaluate 6 months Progression Free Survival (PFS) after early treatment interruption for Grade (G) 3–4 toxicities compared to the control group. Secondary endpoints were to evaluate Time to treatment failure (TTF) and overall survival (OS) in both groups. All statistical analyses were performed using SPSS software (version 19.00, SPSS, Chicago).

**Results:**

18/204 (8.8%) patients had early treatment interruption for serious (G3-G4) irAEs. Early was defined as interruption of IO after only one or two administrations. Immune related nephritis and pancreatitis were the most common irAE that lead to treatment interruption. 6/18 patients received IO-IO combination whereas 12/18 patients antiPD1. In the study group, 12/18 (66.6%) were free from progression at 6 months since IO interruption, TTF was 1.6 months (95% CI 1.6–2.1), mPFS was 7.4 months (95% CI 3.16–11.6) and mOS was 15.5 months (5.1–25.8). In the control group 111/184 (60.3%) patients were free from progression at 6 months, TTF was 4.6 months (95% CI 3.5–5.6), mPFS was 4.6 months (95% CI 3.5–5.6) and mOS was 19.6 months (95% CI 15.1–24.0). In the overall population, mPFS was 5.0 months (95% CI 4.0–5.9) and mOS was 19.6 months (95% CI 15.1–24.0).

**Conclusions:**

ICIs seem to maintain efficacy even after early interruption due to severe irAE.

## Background

Immune-Oncology (IO), alone or in combination, changed the paradigm of treatment of metastatic renal cell carcinoma (mRCC) showing an improvement in Overall Survival (OS) and Progression Free Survival (PFS) compared to standard of care [1–3]. IO induces tumor death in a different way, compared to Vascular Endothelial Growth Factor Receptors (VEGFR)-Tyrosine Kinase Inibitors (TKI), and, consequently, has different adverse events called immune related Adverse Events (irAEs).

Severe irAEs develop in 10–27% of patients treated with anti-Cytotoxic T-Lymphocyte Antigen (CTLA)-4, in amount 12–20% of patients treated with anti-programmed cell death (PD)-1 and 15–20% of patients treated with anti-programmed death-ligand (PD-L)-1 [4–7]. In mRCC G3-G4 irAE develop in 1.7–19% of patients treated with anti-PD1 [8] and 1.3–10.4% of patients treated with anti-PD1 + anti CTLA-4 [1].

Fathal irAE have been reported in almost 0.36% of patients treated with anti PD-1/PDL-1, 1.08% in those treated with anti-CTLA-4 and 1.23% of patients treated with combination [9]. IrAEs can involve kidney, lung, liver and skin but nervous system and osteoarticular manifestation have been described too [10].

According to recent reports, irAEs correlates with a better outcome [11]. IrAE can arise very early or after long time [11]. Long responders’ patients have been reported even after a short time to IO exposure.

Therefore, the aim of our study was to retrospectively evaluate the impact of early interruption of IO treatment, due to irAEs, on outcome of mRCC patients.

## Methods

We retrospectively collected data of mRCC patients treated with IO in 6 Italian referral centers adhering to the Meet-Uro group, between February 2017 and January 2020.

Inclusion criteria were at least 18 years old at the time of enrollment, histological diagnosis of renal cell carcinoma and radiological diagnosis of metastatic disease.

Patients treated with IO as single agent or in combination were considered eligible.

Baseline characteristics were collected at the start of IO. Outcome data, including PFS, TTF, OS and toxicities, were collected too. Data included first line treatment, subsequent IO therapy and previous nephrectomy.

The International Metastatic RCC Database Consortium (IMDC) prognostic risk group was computed at the index date based on the presence of six individual risk factors including time from diagnosis to systemic treatment < 1 year, hemoglobin < lower limit of normal, calcium > 10 mg/dL, platelet > upper limit of normal, neutrophil > upper limit of normal, Performance Status (PS) < 80% (Karnofsky).

IrAEs were graded according to the National Cancer Institute Common Toxicity Criteria for Adverse Events (CTCAE; version 5.0).

Primary endpoint was to evaluate 6 months PFS after treatment interruption for toxicities.

Secondary endpoints were to evaluate OS and Time to Treatment Failure (TTF) in patients. TTF was evaluated as the interval from treatment initiation to premature discontinuation due to cancer progression, adverse events patients’ choice or death. PFS was defined as the time from treatment start to disease progression or death from any cause.

Early interruption was defined as the interruption of ICIs after one or two administration, almost 2 months of treatment.

Patients with no evidence of death were censored at the date of last tumor assessment.

Real-world physician-assessed progression and response was based on clinical criteria or radiographic criteria using Response Evaluation Criteria in Solid Tumors (RECIST) guidelines, with imaging assessments occurring at clinically variable time points.

Baseline demographic and clinical characteristics are described using frequencies and percentages for categorical variables.

Descriptive analysis was made using median values and ranges. All statistical analyses were performed using SPSS software (version 19.00, SPSS, Chicago).

All participating centers received local ethics approval for data collections. The study was conducted in accordance with good clinical practice and the Declaration of Helsinki.

## Results

Data from 204 mRCC patients were retrospectively collected from 6 referral centers. To properly weight the results, patients who did not report early G3-G4 toxicities have been included as control group. Characteristics of patients are described in Table 1. 18/204 (8.8%) patients had early treatment interruption for serious (G3–G4) irAEs, 9/18 after 1 cycle. 10/18 had G3 toxicities whereas 8/18 G4 toxicities.

**Table 1:** Characteristics of patients included in our study. Patients were grouped according to the reason for interruption of ICIs due to irAE or progressive disease

	Interruption of IO	
	G3-G4 irAE 18	PD 186
**Sex**		
Male	10	98
Female	8	38
**Median age**	66.0	60.9
**Site of disease**		
Bone	9	44
Lung	18	73
Lymph nodes	12	69
Liver	6	19
Gland	4	28
**Synchronous metastatic disease**	10	85
**Metachronous metastatic disease**	8	101
**IMDC score**		
Good	1	74
Intermediate	17	99
Poor	0	13
**IO**		
Single agent	12	178
IO–IO combo	6	8
**AID**		
Yes	2	3
Not	16	183
**ECOG PS**		
0	12	96
1	5	72
2	1	18
**Line of treatment**		
1	6	8
2	7	117
3	4	49
Further line	1	12

irAE: immune-relate Adverse Events; G: grade; PD: progressive disease; IMDC score: International Metastatic RCC Database Consortium; AID: autoimmune disease; ECOG PS: Eastern Cooperative Oncology Group Performance Status; IO: Immune-Oncology

In the overall population, 190/204 patients received anti-PD1 whereas 14/204 patients were treated with antiCTLA4+ antiPD1 combination. In the early discontinuation group, 6/18 patients received IO-IO combination whereas 12/18 patients antiPD1 (*p* < 0.0001) (Table 2). In the control group, 6 patients developed G2 irAEs after two month of IO treatment. Characteristic of patients who experienced early irAEs G3-G4 are described in Table 1 whereas Table 2 reports differences between group of patients who interrupted IO due to irAEs and patients who interrupted treatment due to progression.

**Table 2:** Difference between groups according to G3-G4 irAE interruption

	No irAE interruption	irAE interruption	*p*
**PS ECOG**			
0	94	12	0.182
1	72	5	
2	18	1	
**IMDC score**			
Good	55	1	**0.027**
Intermediate	116	17	
Poor	13	0	
**Sex**			
M	129	10	0.154
F	51	8	
**Synchronous metastatic disease**			
Y	85	10	0.448
N	99	8	
**Lung metastasis**			
Y	75	13	**0.014**
N	61	5	
**Liver metastasis**			
Y	19	3	0.759
N	117	15	
**Brain metastasis**			
Y	10	2	0.576
N	126	16	
**Gland metastasis**			
Y	28	2	0.340
N	108	16	
**Peritoneal** **metastasis**			
Y	8	0	0.367
N	176	18	
**IO**			
Anti-PD1	176	12	<** 0.0001**
Anti-PD1+ anti CTLA4	8	6	

Y: YES; N: No; irAEs: immune-related Adverse Events; IO: Immune-Oncology; IMDC: score International Metastatic RCC Database Consortium; ECOG PS: Eastern Cooperative Oncology Group Performance Status; anti-PD1: anti programmed death 1; anti-CTLA4: anti-Cytotoxic T-Lymphocyte Antigen 4

In patients who developed early G3-G4 irAEs and then interrupted treatment, 12/18 (66.6%) patients were free from progression at 6 months from IO interruption whereas, in the control group, patients free from progression at 6 months, were 69/184 (37.5%) *p* 0.1448.

Immune related nephritis and pancreatitis were the most common irAEs that lead to early treatment interruption (Table 3). According to CTCAE version 5.0, nephritis was defined by increase in serum creatinine > 3.0 × baseline, managed initially by stopping nephrotoxic drugs (including over the counter medications), ruling out infection, urinary tract obstruction and correcting hypovolaemia whereas pancreatitis was defined as abdominal pain, pancreatic enzyme elevation and radiological findings of pancreatitis. IrAEs were reversible and were managed mainly with steroids, according to ESMO clinical practice guidelines [6].

**Table 3:** IrAEs that lead to ICIs interruption

IrAE	N
Miositis	1
Diabetes mellitus	1
Ipofisitis	2
Nefritis	3
Pneumonitis	2
Injection reaction	2
Colitis	2
Neuro toxicities	2
Pancreatitis	3
Arthritis	1
Ocular toxicities	1
Hemolytic anemia	1
Hepatitis	2

N: number; IrAE: immune-related adverse events; ICIs: immune checkpoint inhibitors

In patients who interrupted treatment due to early irAEs, TTF was 1.6 months (95% CI 1.6–2.1), mPFS was 7.4 months (95% CI 3.16–11.6) and mOS was 15.5 months (5.1–25.8). In the control group, TTF was 4.6 months (95% CI 3.5–5.6), mPFS was 4.6 months (95% CI 3.5–5.6) and mOS was 19.6 months (95% CI 15.1–24.0).

In the overall population, median PFS was 5.0 months (95% CI 4.0–5.9) and median OS was 19.6 months (95% CI 15.1–24.0) whereas TTF was 4.1 months (95% CI 3.2–4.9). 125/204 patients were free from progression at 6 months.

12/18 patients did not receive further treatment after IO due to clinical deterioration.

## Discussion

IrAEs contribute to mortality and morbidity in mRCC and represent a relevant issue for healthcare system. Symptoms and signs are often insidious and similar to cancer related symptoms so that irAEs represent a clinical challenge for physicians.

Biological biomarkers for irAEs are mostly unknown [12]. Baseline circulating Interleukin (IL)-17 was related to G3-G4 colitis [13] whereas Gowen et al. reported a different baseline profiling of antibodies in patients who developed irAEs [14].

In clinical practice, pre-existing organ insufficiency is supposed to be associated to higher risk to develop irAEs as well as pre-exiting autoimmune disease (AID) [15]. In patients treated with ipilimumab, AID exacerbated in 27% of cases with 33% of irAE [16] whereas in patients treated with anti-PD-1, 38% had a flare of a preexisting AID with 10% of G3-G4 irAEs and a discontinuation rate of 4% [17–19]. In a larger cohort of patients, Tison et al. reported AID flare in 47% of cases and irAEs in 43% with a discontinuation rate of 21% [20]. Recrudescence of AID and irAEs are usually managed with corticosteroids. The use of immunosuppressive therapy at initiation of IO is associated to fewer irAEs compared to patients who did not receive corticosteroids.

In our real-world analysis, the incidence of early G3-G4 irAEs was almost 10–15%.

Outcomes here reported, demonstrate the efficacy of IO even after early interruption due to irAEs. Furthermore, after 6 months from treatment interruption, 66.6% of patients were free from progression demonstrating long-term benefit from IO. Indeed, G3-4 irAEs seem to be related to efficacy of IO even when treatment is early interrupted due to toxicities [11]. Differences in mPFS and mOS between the irAEs group and the control group was not analyzed due to the small sample size and the heterogeneity of patients in groups but the ones who experienced severe irAEs tend to a longer mPFS. Indeed, we reported longer mPFS in severe early irAEs group compared to mPFS reported in the pivotal trials of ICIs and other real-world experiences of IO in RCC. Median OS here reported was comparable to previous reports.

The hypothesis underling brilliant response after the onset of irAEs is that an exuberant activation of immune system against our self-tissues becomes an exuberant response against tumor tissue [11, 15]. Several modification have been seen in the immune system of patients treated with IO, which can explain long-term response [21]. In vitro, anti-CTLA4 is associated to an increase expansion and enhance effector function of memory CD8+ T cell, which can be related to long-term response [22]. Instead, it is uncertain if anti-PD1 increases CD8+ effector memory cells in cancer patients. Indeed, as reported in vitro, anti-PD1 enhance cytokine production in human T-cells and, in a T-reg suppression assay, it completely restored CD4+ T-responder cell proliferation and partially restored IFNγ production [23].

It is reasonable to suppose that in patients who develop early G3-4 irAEs, ICIs promote adapted states of hyper-responsiveness in immune cells, which promote anti-tumor immunity. This subgroup of patients might have a “hyper-immune system” and early G3–G4 irAEs could represent a predictive parameter for persistent adaptations in the immune system, which can persist and display memory-like features. Limits of our study are the small sample size, the retrospective collection of data and the lack of central radiological review. A prospective validation is needed to straighten these findings.

## Conclusions

Pathogenesis and treatment of irAEs represent an interesting research field as it remains under debate. Interruption is often required after irAEs, with consequent doubts about treatment efficacy. Nevertheless, our study, with the limit of a retrospective collection, confirms that in mRCC patients, even after early interruption due to irAEs, IO maintain clinical and radiological efficacy.

## Data Availability

Authors declare that data and material collected are available after properly request.

## Abbreviations

irAE: Immune-related Adverse Events
G: Grade
PD: Progressive disease
IMDC: International Metastatic RCC Database Consortium
AID: Autoimmune disease
ECOG PS: Eastern Cooperative Oncology Group Performance Status
IO: Immune-Oncology
OS: Overall Survival
PFS: Progression Free Survival
TTF: Time To Treatment Failure
mRCC: Metastatic Renal Cell Carcinoma
MSKCC: Memorial Sloane Kattering Cancer Center
RECIST: Response Evaluation Criteria in Solid Tumors
ICIs: Immune-Checkpoint Inhibitors
(CTLA)-4: Anti-Cytotoxic T-Lymphocyte Antigen
(PD)-1: Anti-Programmed Cell Death
(PD-L)-1: Anti-Programmed Death-Ligand
